# Changes in CO_2_-Derived Variables, Induced by Passive Leg Raising Test, Detect Preload Responsiveness in Mechanically Ventilated Patients: A Pilot Study

**DOI:** 10.3390/jcm15041551

**Published:** 2026-02-15

**Authors:** Angeliki Baladima, Stelios Kokkoris, Dimitrios Tzalas, Konstantina Kolonia, Theodora Ntaidou, Theodoros Pittaras, Athanasios Trikas, Ioannis Vasileiadis, Christina Routsi

**Affiliations:** 1 First Department of Critical Care Medicine and Pulmonary Services, Medical School, National and Kapodistrian University of Athens, Evangelismos Hospital, 45-47 Ipsilantou Street, 106 76 Athens, Greece; angiebalad@gmail.com (A.B.); stkokkoris@med.uoa.gr (S.K.); kon.kolonia@gmail.com (K.K.); theodorantaidou@yahoo.com (T.N.); ioannisvmed@yahoo.gr (I.V.); 2 Department of Cardiology, Evangelismos Hospital, 106 76 Athens, Greece; dimtzalas@gmail.com (D.T.); atrikas@otenet.gr (A.T.); 3 Haematology Laboratory-Blood Bank, Aretaieion Hospital, National and Kapodistrian University of Athens, 115 28 Athens, Greece; tpittaras@med.uoa.gr

**Keywords:** central venous-to-arterial carbon dioxide partial pressure difference, passive leg raising, PLR, fluid responsiveness, circulatory shock, CO_2_-derived markers, velocity-time integral, VTI

## Abstract

**Background/Objectives.** Changes in CO_2_-derived variables during a fluid challenge have been proposed as markers of fluid responsiveness. We investigated whether, instead of fluid administration, passive leg raising (PLR)-induced changes in the CO_2_-derived variables, namely central venous-arterial carbon dioxide partial pressure (P(cv-a)CO_2_) and the ratio between P(cv-a)CO_2_ and the arterial-central venous oxygen content (P(cv-a)CO_2_/C(a-cv)O_2_), could detect preload responsiveness in critically ill patients. **Methods.** We studied 30 mechanically ventilated patients in whom a PLR test was performed due to acute circulatory failure. Routine hemodynamic variables, velocity-time integral (VTI), in the left ventricular outflow tract, and CO_2_-derived variables, were measured before, during, and after a PLR test. A PLR-induced increase in VTI of ≥10% defined preload responsiveness. The differences (Δ) of P(cv-a)CO_2_ and P(cv-a)CO_2_/C(a-cv)O_2_ between PLR and pre-PLR were calculated. The predictive values of PLR-induced changes in the CO_2_-derived variables was determined by receiver operating characteristic area under curves (ROC-AUCs). **Results.** Fifteen patients (50%) were classified as preload responsive. ΔP(cv-a)CO_2_ and ΔP(cv-a)CO_2_/C(a-cv)O_2_ were correlated with VTI changes and differed significantly between responders and non-responders −1.3 (−2–−0.6) vs. 0.6 (−0.1–1.1) mmHg, *p* < 0.001, and −0.38 (−0.97–−0.34) vs. 0.1 (−0.15–0.57) mmHg/mL O_2_, *p* < 0.001, respectively. The PLR-induced decrease in P(cv-a)CO_2_ was significantly associated with preload responsiveness (OR 0.48, CI 0.20–0.89, *p* = 0.016, bootstrap CI 0–0.85). The AUC curves for both ΔP(cv-a)CO_2_ and ΔP(cv-a)CO_2_/C(a-cv)O_2_ ratio to predict preload responsiveness were 0.89 (CI 0.74–1), *p* < 0.001, and 0.85 (CI 0.70–1), *p* < 0.001, respectively. **Conclusions.** In mechanically ventilated ICU patients with circulatory shock, PLR-induced changes in P(cv-a)CO_2_ and P(cv-a)CO_2_/C(a-cv)O_2_ ratio were correlated with VTI changes. The change in P(cv-a)CO_2_ was the only variable detecting preload responsiveness assessed by PLR; therefore, it could serve as an indirect marker, useful to guide fluid resuscitation when cardiac output measurement is not feasible.

## 1. Introduction

In the setting of critical care, assessing cardiac preload responsiveness is paramount to avoid the administration of fluids to patients who will not benefit [1]. However, evaluating the preload responsiveness in the intensive care unit (ICU) remains a clinical challenge. Static measures of volume status, such as blood pressure, cardiac filling pressures, heart rate, cardiac output, mixed venous oxygen saturation, or inferior vena cava (IVC) diameter variation, are neither sensitive nor specific, offering limited utility for predicting the hemodynamic response to fluid administration [1,2]. Current practice guidelines [1] recommend the use of dynamic functional tests to assess preload responsiveness, such as a fluid challenge or passive leg raising (PLR)-induced stroke volume changes, among others (e.g., pulse pressure variation or stroke volume variation) [1,2,3,4,5,6,7,8]. By identifying patients whose cardiac output increases after a fluid bolus or after a PLR test, clinicians can tailor fluid administration to improve perfusion while preventing fluid overload. However, special devices are required to measure the evolution of the above-mentioned hemodynamic variables during a dynamic test.

As it has been previously demonstrated, the changes in stroke volume can be assessed non-invasively using transthoracic echocardiography (TTE), by measurement of changes in left ventricular outflow tract (LVOT) velocity-time integral (VTI), itself being a surrogate of left ventricular stroke volume [9,10].

On the other hand, CO_2_-derived variables, namely central venous minus arterial carbon dioxide partial pressures (P(cv-a)CO_2_) and the ratio of P(cv-a)CO_2_ to the arterial minus central venous oxygen contents (C(a-cv)O_2_) have gained particular attention as metabolic indices, useful to assess the adequacy of cardiac output and global tissue perfusion respectively [11,12,13,14]. An increased P(cv-a)CO_2_ value (>6 mmHg) represents a low cardiac output [11,12,13] whereas for the P(cv-a)CO_2_/C(a-cv)O_2_ ratio, which serves as a surrogate of the respiratory quotient, a value of more than 1.4 has been considered abnormal, indicating inequivalence between global CO_2_ production and oxygen consumption, i.e., anaerobic metabolism [12].

An earlier study, by Mecher et al. [15], had shown that a fluid bolus decreased high P(cv-a)CO_2_ in patients with septic shock and low cardiac output. Since then, a number of studies in critical care settings demonstrated that fluid administration can cause a decrease in the P(cv-a)CO_2_ due to an increase in cardiac output [16,17,18,19,20]. However, almost all the available data come from studies focusing on the P(cv-a)CO_2_ change after a fluid challenge with a fluid bolus. We hypothesized that, instead of a fluid bolus, the PLR, as a safe and reversible test, could also detect preload responsiveness through changes in the CO_2_-derived variables. To this end, we investigated whether PLR-induced changes in CO_2_-derived variables could detect PLR responsiveness, assessed by TTE, in mechanically ventilated patients with circulatory shock.

## 2. Methods

This is a prospective, observational clinical study conducted in the multidisciplinary ICU at Evangelismos Hospital, Athens, Greece, a tertiary medical center. The study was conducted in accordance with the Declaration of Helsinki, and the protocol was approved by the Ethics Committee of Evangelismos Hospital (number 285/2017.13.11), followed by a protocol revision (number 495/2025.17.12). No intervention was required since all measurements were decided by the attending physician as part of the routine use of the PLR test, following the standard protocols of care for patients with shock. Therefore, the requirement for written informed consent was waived, and only oral informed consent was obtained from the patient’s next of kin.

### 2.1. Patients

ICU patients aged 18 years and older, undergoing mechanical ventilation in a volume-controlled mode, sedated, with circulatory shock and need for a PLR test, as decided by the attending physician, were eligible to participate in the study. Exclusion criteria were: contraindication to PLR maneuver (i.e., intracranial hypertension, intra-abdominal hypertension, lower extremity or spinal injury), echocardiography examination with poor echogenicity, and lack of an arterial and/or a central venous line placed in an internal jugular or subclavian vein at the time of the PLR test. Patients with cardiac arrhythmias, non-sinus rhythm, atrioventricular conduction abnormalities, paced rhythm, and significant valvulopathies were also excluded (because these can affect the Doppler parameters used to assess the PLR response).

Circulatory shock was defined as hypotension (systolic blood pressure < 90 mmHg and/or mean arterial pressure < 65 mmHg), persisting despite adequate volume resuscitation, requiring administration of vasoactive agents [21]. Septic shock was defined based on the Sepsis-3 criteria [22], and the patients were categorized as septic and non-septic shock. Demographic data, anthropometric characteristics, admission diagnosis and illness severity, evaluated by the Sequential Organ Failure Assessment (SOFA) score [23] on the day of the study were collected. A brief characterization of the cohort in terms of ARDS severity and dead-space ventilation is described in the Supplementary Materials.

### 2.2. Protocol and Measurements

**Figure 1:**
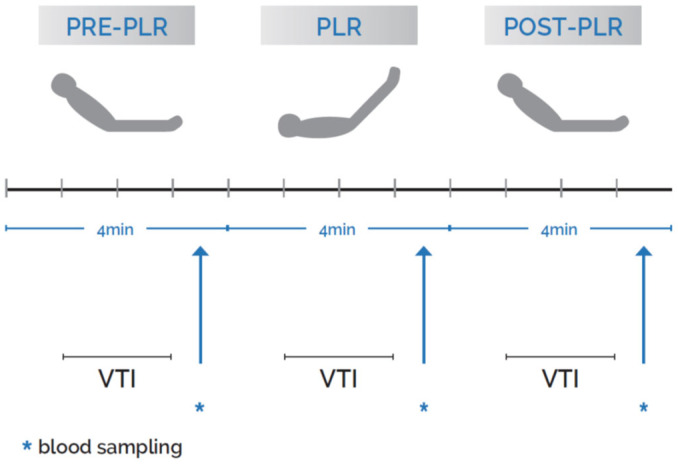
Study protocol. PLR, VTI, velocity-time integral.

The protocol sequence is shown in Figure 1. First, with the patient in the initial semi-recumbent position, hemodynamic variables including heart rate, invasive arterial pressure, and central venous pressure (CVP) were obtained. Then the IVC was assessed through the subcostal view and its diameter was measured perpendicularly to the vessel at approximately 1 cm from its junction with the right atrium. The maximum IVC diameter throughout the respiratory cycle was used. Ventilator settings, respiratory parameters, vasopressors, and/or inotropic use were also recorded.

Subsequently, the VTI was calculated using a phased-array transducer with cardiac settings. Pulse wave Doppler mode was employed in the LVOT through the apical five-chamber cardiac view, while taking care to align the ultrasound beam with the blood flow in the LVOT as much as possible. Three consecutive velocity curves were obtained [24], the outline of each individual curve was traced to determine its VTI value, and the average VTI was calculated.

A PLR test was then performed as described elsewhere [7,24,25,26]. Briefly, PLR was performed using an automatic elevation bed by raising the patient’s lower limbs to a 45° angle to the horizontal plane while the patient’s trunk was lowered from a semi-recumbent to supine position with no changes in the hip angle.

Measurements were obtained at the following three time intervals: just prior, during, and after a PLR maneuver. The first VTI was obtained with the patient at the initial semi-recumbent position, as described above (pre-PLR VTI), a second measurement was obtained during a PLR maneuver (PLR-VTI), and a third measurement of VTI after the patient was returned back to the initial semi-recumbent position (post-PLR VTI). Each time interval lasted for 4 min (Figure 1).

TTE examination was conducted using a Vivid E9 echocardiograph (GE Healthcare, Chicago, IL, USA), which was performed by an experienced cardiologist (A. B.) who was not involved in the management of patients and was blinded to the clinical status and the gas exchange measurements.

PLR responsiveness was assessed by changes in the VTI measurements. Change in VTI (ΔVTI) was defined as [(PLR-VTI minus pre-PLR VTI)/pre-PLR VTI] × 100%. Patients in whom the PLR test resulted in a VTI increase of ≥10% were classified as PLR responders [2,10,17,18,19,20,21,22,23,24,25,26,27,28] and the remaining ones as PLR non-responders.

The IVC and CVP values were recorded at baseline, whereas heart rate, invasive arterial pressure, and arterial and central venous blood gases were recorded at each time interval. The dosage of the vasoactive drugs as well as the ventilator settings remained unchanged during the procedure.

### 2.3. Intra-Observer Variability

Intra-observer variability of VTI measurements was assessed in 24 subjects. Paired measurements demonstrated minimal bias, with a mean difference of −0.14 (CI: −0.68–0.40) cm. The intraclass correlation coefficient was 0.968. The standard error of measurement was 0.89 cm. The within-subject coefficient of variation was 5.5%, and the 95% limits of agreement ranged from −2.63 to 2.35 cm, according to the Bland–Altman test, Figure S1. The combination of minimal bias, narrow limits of agreement, and a high intraclass correlation coefficient implies increased intra-observer reliability.

### 2.4. Gas Exchange Measurements

Immediately after TTE and recording of the hemodynamic and ventilator parameters, two samples of blood were simultaneously drawn: one from the arterial catheter and one from the distal port of the internal jugular or subclavian catheter. Each sample was drawn in 2.5 mL heparinized syringes after discarding the first 2 mL of blood and was analyzed for arterial and central venous blood gases (ABL 300, Radiometer, Copenhagen, Denmark).

The following variables were recorded: partial pressure of arterial oxygen (PaO_2_) and arterial carbon dioxide (PaCO_2_), partial pressure of central venous oxygen (PcvO_2_) and central venous carbon dioxide (PcvCO_2_), hemoglobin arterial oxygen saturation (SaO_2_) and central venous oxygen saturation (ScvO_2_), as well as hemoglobin concentration and arterial lactate levels.

CO_2_-derived variables were calculated as follows: The arterial oxygen content (CaO_2_) and the central venous oxygen content (CcvO_2_) were calculated using the following formulas: CaO_2_ = (SaO_2_ × Hb × 1.34) + (0.0031 × PaO_2_) and CcvO_2_ = (ScvO_2_ × Hb × 1.34) + (0.0031× PcvO_2_), respectively. Then, the difference between them (arterial-central venous oxygen content difference, C(a-cv)O_2_) as well as the P(cv-a)CO_2_) and the P(cv-a)CO_2_/C(a-cv) O_2_ ratio were calculated.

### 2.5. Statistical Analysis

Continuous variables were expressed as median with interquartile range (IQR) and categorical variables as absolute numbers and percentages. Comparisons between two independent groups were performed using the Mann–Whitney U test. For repeated measurements of continuous variables across time points, the Friedman test was applied, followed by post hoc pairwise comparisons with appropriate Bonferroni corrections for multiple testing. Correlations between variables were calculated with Spearman’s rho correlation coefficient. Intra-observer variability was estimated by the intraclass correlation coefficient, standard error of measurement, coefficient of variation, and Bland–Altman test. Association between clinically meaningful markers and fluid responsiveness was further explored using a multivariate logistic regression model, and results were reported as odds ratios (ORs) with corresponding 95% confidence intervals (CIs).

However, the model produced wide uncertainty due to the small sample size. Therefore, to account for the small sample size, multivariable logistic regression was performed using Firth’s penalized-likelihood approach. The primary model included two clinically selected variables (CVP, lactate). The rationale behind the selection of those two particular variables is mainly based on clinical grounds: lactate is a surrogate of circulatory shock, while CVP is a measure of venous return, which could potentially affect cardiac performance. Model coefficients were internally validated using non-parametric bootstrap resampling with 1000 iterations, and percentile-based bootstrap CIs were calculated. ORs were obtained by exponentiating penalized regression coefficients. Model calibration was evaluated graphically using a nonparametric Loess-smoothed calibration plot comparing predicted probabilities with observed outcomes, alongside numerical assessment using the calibration slope with bootstrap internal validation.

Receiver operating characteristic (ROC) curve analysis was used to assess the predictive performance of individual markers and models, with the area under the curve (AUC) reported. Cut-off values were estimated by Youden’s index. A two-sided *p*-value < 0.05 was considered statistically significant. All statistical analyses were performed using SPSS version 24 (IBM Corp., Armonk, NY, USA) and R version 4.1.1 (Vienna, Austria).

## 3. Results

### 3.1. Patient Characteristics

Thirty patients were enrolled in the study. Their median age was 59 (IQR 54–75) years, and 23 (77%) were males. On the study day, the median SOFA score was 10 (9–14), arterial lactate 1.9 (1–3.1) mmol/L, and the CVP and IVC diameters were (9–14) mmHg and 20 (17–23) mm, respectively. Admission diagnosis was medical in 18, surgical in 10, and trauma in the remaining 2 patients.

**Figure 2:**
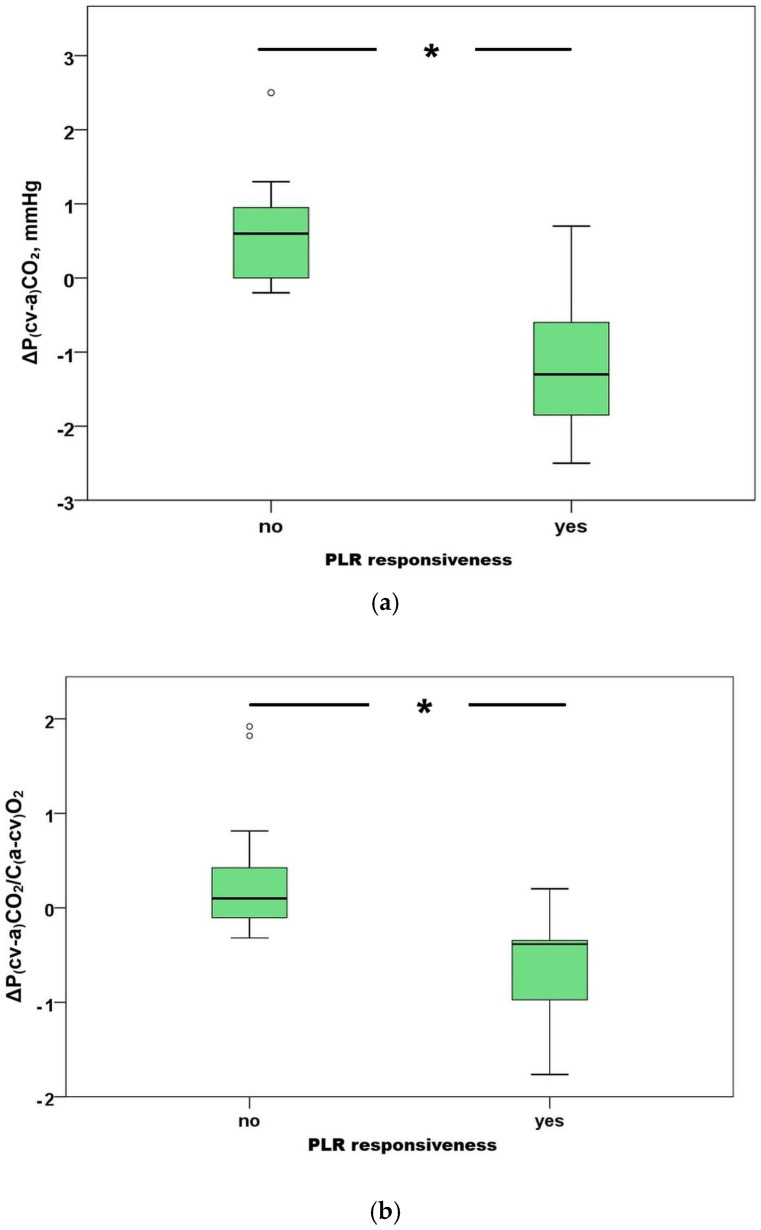
Boxplots of (**a**) ΔP(cv-a)CO_2_ and (**b**) ΔP(cv-a)CO_2_/C(a-cv)O_2_ according to PLR responsiveness, * *p* < 0.001. Abbreviations: Δ, difference between PLR and pre-PLR; P(cv-a)CO_2_, central venous minus arterial carbon dioxide partial pressure; C(a-cv)O_2_, arterial minus central venous oxygen content.

Baseline (i.e., pre PLR) P(cv-a)CO_2_ and P(cv-a)CO_2_/C(a-cv)O_2_ ratio were 6.4 (5.1–7.4) mmHg and 2.4 (2.0–2.8), respectively. Fifteen patients (50%) were classified as PLR responsive. Table 1 shows the baseline characteristics of patients grouped by responsiveness status. Of all parameters studied, only ΔP(cv-a)CO_2_ and ΔP(cv-a)CO_2_/C(a-cv)O_2_ ratio were significantly different between responders and non-responders, −1.3 (−2–−0.6) vs. 0.6 (−0.1–1.1) mmHg, *p* < 0.001, and −0.38 (−0.97–−0.34) vs. 0.1 (−0.15–0.57) mmHg/mlO_2_, *p* < 0.001, respectively (Figure 2a,b).

### 3.2. Correlations

**Figure 3:**
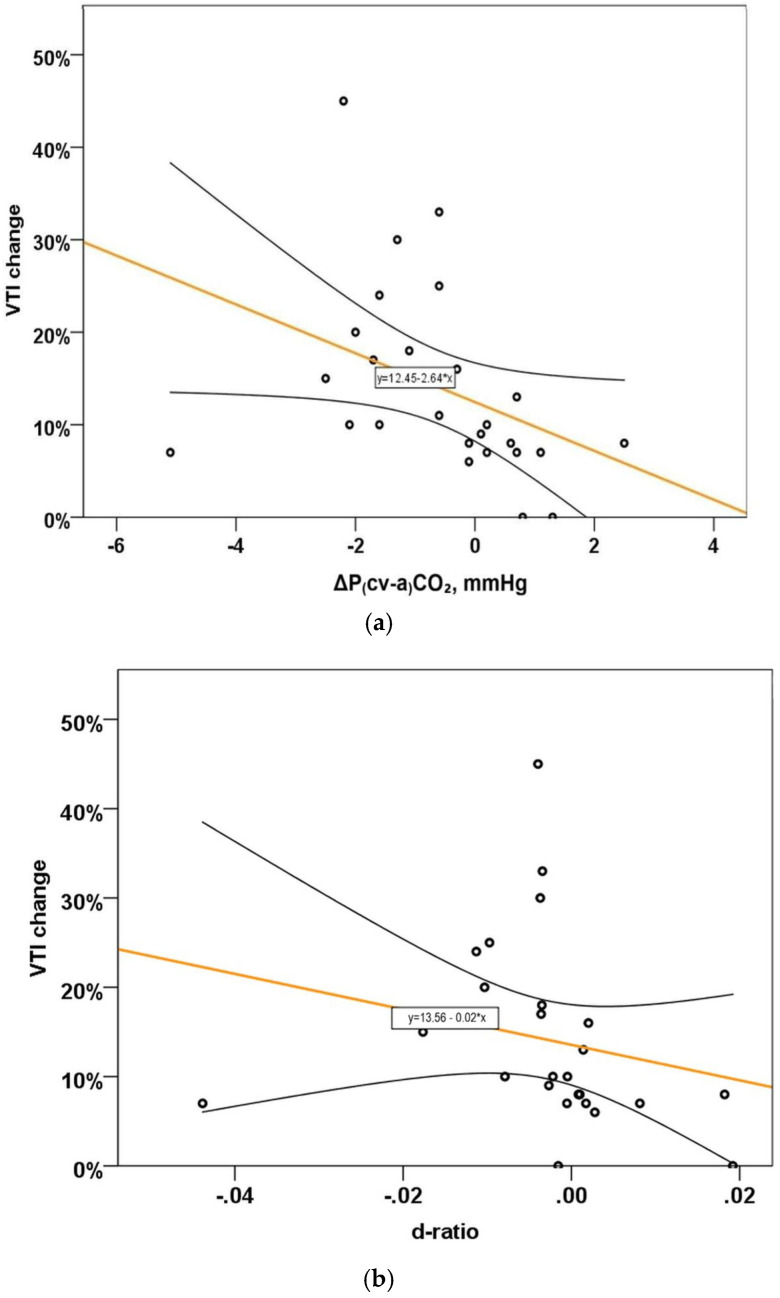
Scatterplots of (**a**) ΔP(cv-a)CO_2_ and (**b**) ΔP(cv-a)CO_2_/C(a-cv)O_2_ with VTI change (%). Abbreviations: PLR, passive leg raising; VTI, velocity-time integral; Δ, difference between PLR and pre-PLR; P(cv-a)CO_2,_ central venous minus arterial carbon dioxide partial pressure; C(a-cv)O_2_, arterial minus central venous oxygen content.

Changes in both P(cv-a)CO_2_ and the P(cv-a)CO_2_/C(a-cv)O_2_ ratio between pre- and during PLR were significantly correlated with the change in VTI (Spearman’s rho = −0.60, *p* < 0.001, and 0.58, *p* < 0.001, respectively), as shown in Table 2. Figure 3a,b show that the changes in both P(cv-a)CO_2_ and P(cv-a)CO_2_/C(a-cv)O_2_ ratio were inversely correlated with the change in VTI, i.e., a decrease in either of them was significantly correlated with an increase in VTI.

### 3.3. Dynamics of CO_2_-Derived Variables Across the PLR Test

**Figure 4:**
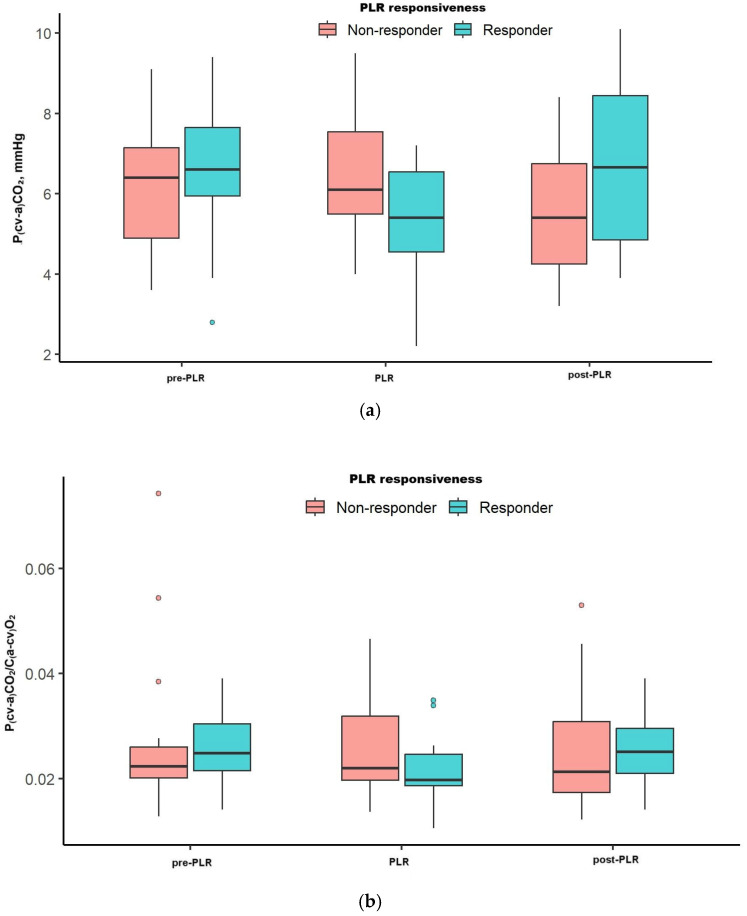
Boxplots showing the kinetics of (**a**) P(cv-a)CO_2_ and (**b**) P(cv-a)CO_2_/C(a-cv)O_2_ over the PLR test. The overall trend over time was estimated with Friedman test, while the between-groups comparisons were performed by Mann–Whitney test. Overall trend over time as well as between-group comparisons was non-significant for both markers. Abbreviations: P(cv-a)CO_2,_ central venous minus arterial carbon dioxide partial pressure; C(a-cv)O_2_, arterial minus central venous oxygen content; PLR, passive leg raising.

Figure 4a,b and Table 3 show the kinetics of P(cv-a)CO_2_ and the P(cv-a)CO_2_/C(a-cv)O_2_ ratio before, during, and after the PLR maneuver. Overall trend over time, as well as between-group comparisons, were non-significant for both markers (Figure 4, Table 3). In contrast, VTI significantly increased during the PLR test and returned to the baseline [from 14.2 (13–17.4) cm increased to 17.0 (15.5–19.1) cm and dropped to 14.8 (12.5–18.0) cm, *p* < 0.001], Table 3. Similarly, MAP significantly increased during the PLR test [from 77 (73 to 83) to 84 (75–90) mmHg] and returned to 77 (70–86) mmHg, *p* < 0.001], Table 3

### 3.4. Multivariate Logistic Regressions

In Firth’s penalized logistic regression analysis including CVP, lactate, and ΔP(cv-a)CO_2_, the latter was significantly associated with preload responsiveness, with higher values associated with lower odds of the outcome (OR 0.48, CI 0.20–0.89). CVP and lactate were not significantly associated with the outcome. Penalized regression was used to account for the limited sample size and reduce small-sample bias. Bootstrap analyses were added for internal validation; while CIs were wider due to the small sample size, results were directionally consistent with the penalized regression model and confirmed the robustness of the association between P(cv-a)CO_2_ and the outcome, Table 4. On the contrary, the difference between PLR and pre-PLR P(cv-a)CO_2_/C(a-cv)O_2_ ratio was not significantly associated with fluid responsiveness (OR = 0.0001, *p* = 0.29). The bootstrap-validated calibration slope had a median value of 1.42 (CI: 1.21–41.99). While values below 1—indicative of overfitting—were not observed, the wide confidence interval reflects substantial uncertainty and limited precision due to the small sample size. In addition to that, graphical assessment using a Loess-smoothed calibration plot demonstrated good agreement between predicted and observed risks at low predicted probabilities, with underestimation of risk in the intermediate probability range and instability at extreme predicted probabilities, reflecting sparse data in these regions (Figure S2).

#### Sensitivity Analyses

In a multivariable Firth penalized logistic regression including CVP, lactate, ΔP(cv-a)CO_2_, and norepinephrine dose, ΔP(cv-a)CO_2_ was significantly associated with the preload responsiveness. Specifically, higher ΔP(cv-a)CO_2_ values were associated with lower odds of the outcome (OR 0.50, CI 0.21–0.92, *p* = 0.023), whereas CVP, lactate, and norepinephrine dose were not, Table 4. In another model including shock category (septic versus non-septic shock) instead of norepinephrine dose, ΔP(cv-a)CO_2_ remained significantly associated with the outcome (OR 0.49, CI 0.20–0.90, *p* = 0.018). In both sensitivity analyses, bootstrap CIs were wider but directionally consistent (Table 4).

### 3.5. Predictive Value of PLR-Induced ΔP(cv-a)CO_2_ and ΔP(cv-a)CO_2_/C(a-cv)O_2_ for Preload Responsiveness

**Figure 5:**
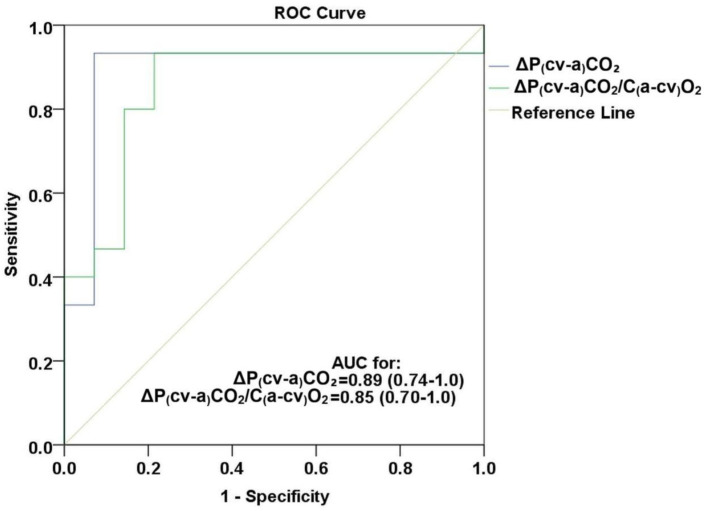
ROC curves for P(cv-a)CO_2_ and ΔP(cv-a)CO_2_/C(a-cv)O_2_ for prediction of fluid responsiveness. Abbreviations: ROC, receiver operating characteristic; AUC, area under curve; Δ, difference between PLR and pre-PLR; P(cv-a)CO_2_, central venous minus arterial carbon dioxide partial pressure; C(a-cv)O_2_, arterial minus central venous oxygen content.

A ROC analysis revealed that a decrease in both P(cv-a)CO_2_ and P(cv-a)CO_2_/C(a-cv)O_2_ ratio during the PLR test was a good predictor for preload responsiveness with AUCs of 0.89 (CI 0.74–1), *p* < 0.001, and 0.85 (CI 0.70–1), *p* < 0.001, respectively, Figure 5.

Using a threshold of −0.25 for ΔP(cv-a)CO_2_, sensitivity and specificity were both 93.3% (CI 68.1–99.8). Positive and negative predictive values were also 93.3%. The positive likelihood ratio was 14.0 (CI 2.09–44.7), and the negative likelihood ratio was 0.07 (CI 0.02–0.48), Table 5. A threshold of −0.003 for ΔP(cv-a)CO_2_/C(a-cv)O_2_ yielded a sensitivity of 78.6% (CI 49.2–95.3), and a specificity of 93.3% (CI 68.1–99.8). The positive predictive value was 91.7% (CI 61.5–99.8), and the negative predictive value was 82.4% (CI 56.6–96.2). The positive likelihood ratio was 11.8 (CI 1.74–38.5), while the negative likelihood ratio was 0.23 (CI 0.10–0.65), Table 5. Confidence intervals were wide, particularly for likelihood ratios, reflecting the small sample size; therefore, these estimates should be considered exploratory and hypothesis-generating.

## 4. Discussion

The main findings of the present study can be summarized as follows: in critically ill patients with circulatory shock undergoing mechanical ventilation, (i) a PLR test-induced increase in VTI, indicating preload responsiveness, was associated with a decrease in both P(cv-a)CO_2_ and P(cv-a)CO_2_/C(a-cv)O_2_ ratio, (ii) the PLR-induced decrease in P(cv-a)CO_2_ was the only factor detecting preload responsiveness.

The inverse correlation that was observed between changes in VTI and the CO_2_-derived variables induced by the PLR test seems plausible and in accordance with the existing knowledge. A number of studies thus far have investigated the changes in the CO_2_-derived variables, particularly of the P(cv-a)CO_2_, after volume expansion via fluid administration [16,17,18,19,20,29,30]. They have shown that, in fluid-responsive critically ill patients, a fluid bolus is associated with a decrease in P(cv-a)CO_2_.

Indicatively, Pierrakos et al. [18] have shown that a fluid bolus decreased the P(cv-a)CO_2_ levels in critically ill patients with abnormal P(cv-a)CO_2_ (i.e., >6 mmHg), whereas a correlation between changes in cardiac index and changes in P(cv-a)CO_2_ was observed in those patients who had a low cardiac index before the fluid bolus [18]. In that study, the amount of the given fluid bolus was not reported. Zhou et al. [30] have recently shown that the difference in P(cv-a)CO_2_ before and immediately after a fluid challenge of 500 mL of Ringer’s solution is a potential indicator of fluid responsiveness in mechanically ventilated patients with circulatory shock.

Similarly, in a recent post hoc analysis of a multicenter prospective study in mechanically ventilated patients with acute circulatory failure, by Mallat et al. [19], the changes in P(cv-a)CO_2_ during volume expansion by 500 mL of crystalloid solution could assess fluid responsiveness. Specifically, a post-fluid challenge decrease in P(cv-a)CO_2_ ≤ 2.1 mmHg effectively differentiated fluid responders from fluid non-responders, with a positive predictive value of 90% and a negative predictive value of 58%. Additionally, in accordance with our findings, the change in P(cv-a)CO_2_ was independently associated with fluid responsiveness in multivariable analysis. Notably, in our study, although changes in both P(cv-a)CO_2_ and P(cv-a)CO_2_/C(a-cv)O_2_ ratio were correlated with changes in VTI, in the multivariable analysis, only the P(cv-a)CO_2_ changes were independently associated with PLR responsiveness.

The PLR test serves as a “self-volume infusion” of approximately 300 mL of fluid [2].

Notably, when using the semi-recumbent starting position, which was followed in the present study, the PLR maneuver induces a larger increase in cardiac preload compared with the supine starting position and may be preferred for predicting PLR responsiveness [26].

The present study adds to the existing knowledge the complementary information that the PLR test could be used instead of a fluid bolus, as performed in the above-mentioned studies, to assess the effectiveness of CO_2_-derived variables in detecting preload responsiveness. Furthermore, since PLR, by shifting venous blood from the legs to the intrathoracic compartment, acts like a transient and reversible volume expansion (in contrast to the permanent effect of a bolus fluid infusion), we were able to assess the evolution of the hemodynamic and metabolic indices after the PLR maneuver. As expected, the changes in VTI and CO_2_-derived variables during the PLR test returned to the baseline values after the test, confirming the temporary effect of the PLR on these variables.

Although changes in the VTI during a PLR for predicting preload responsiveness have already been studied [2,25,28], the concomitant changes in the CO_2_-derived variables during the maneuver have not been included so far.

Interestingly, PLR changes in VTI in combination with end-tidal carbon dioxide (which is known to be related to native cardiac output) successfully predicted fluid responsiveness in patients supported with veno-arterial extracorporeal membrane oxygenation (VA ECMO) [31]. Due to complex modifications of the PO_2_ and PCO_2_ during the passage of blood through the ECMO circuit, CO_2_-derived variables are not widely monitored during ECMO. As a result, information on the P(cv-a)CO_2_ and the P(cv-a)CO_2_/C(a-cv)O_2_ ratio in VA ECMO patients is scarce [32,33]. In a study evaluating the CO_2_-derived variables, in blood samples drawn from the venous entry and arterial exit of ECMO, P(cv-a)CO_2_ and P(cv-a)CO_2_/C(a-cv)O_2_ ratio were higher in the early death group in patients with cardiogenic shock on VA ECMO [32]. Accordingly, a recent review underscoring the need for high-quality evidence regarding the optimal fluid strategy in ECMO-supported patients recommends (among other techniques) that an elevated P(cv-a)CO_2_ > 6 mmHg could be potentially valid as a target of fluid resuscitation in ECMO patients [34]. However, though interesting parameters in this scenario, the application of CO_2_-derived variables in ECMO as a hemodynamic goal for therapy or as an additional marker of patient severity is currently unclear [32].

The ability of the changes in the CO_2_-derived variables, especially of the P(cv-a)CO_2_, before and during a PLR test, to detect cardiac preload responsiveness, as shown in the present study, implies practical implications. Since P(cv-a)CO_2_ can be routinely measured from blood samples, its change during a PLR maneuver could serve as a surrogate of the VTI change, and thus, for the evaluation of PLR response. This could be useful in everyday clinical practice, especially in the absence of cardiac output measurements, to guide the fluid administration in the setting of critical care, in order to avoid unnecessary and potentially harmful fluid loading.

### Limitations

A first limitation of our single-center study is that it included a small sample size of ICU patients, which could compromise our results. Therefore, this study should be considered a pilot, exploratory study, and its results should be considered hypothesis-generating and need external validation. Second, the PLR effects on stroke volume were assessed only non-invasively by TTE examination and calculation of the VTI, and not by a direct measurement of cardiac output, considered the gold standard. As it has been previously shown, a weak correlation between VTI and stroke volume obtained by invasive monitoring methods might exist [35], partially explained by the small changes in stroke volume induced by a PLR maneuver [36]. However, it has been shown that after rapid administrations of 100 mL hydroxyethyl starch, a ΔVTI > 10% predicted fluid responsiveness with a sensitivity and specificity of 95% and 78%, respectively, in patients with acute circulatory failure and mechanical ventilation with low tidal volume [37]. In addition, from a practical point of view, it should be noted that VTI is routinely estimated as part of the hemodynamic monitoring in contemporary ICUs. Third, though ventilator settings, vasoactive agents, and sedation were kept unchanged during the PLR, various confounding factors such as V/Q mismatching and subtle changes in metabolic factors cannot be excluded. Finally, venous and arterial CO_2_ content difference, in order to overcome the Haldane effect, which has been described as a potential cause of an increased P(cv-a)CO_2_ value [38], was not measured.

## 5. Conclusions

In summary, in mechanically ventilated ICU patients with circulatory shock, PLR-induced changes in P(cv-a)CO_2_ and P(cv-a)CO_2_/C(a-cv)O_2_ ratio were correlated with VTI changes. The change in P(cv-a)CO_2_ was the only factor detecting preload responsiveness assessed by PLR; therefore, this variable could serve as an indirect marker, useful to guide fluid resuscitation when cardiac output measurement is not feasible. Larger studies are warranted to confirm these findings.

## Figures and Tables

**Table 1:** Patients’ baseline characteristics and PLR- induced changes in CO_2_-derived variables according to responsiveness status.

Variable	All Patients *n* = 30		Non-Responders *n* = 15		Responders * n = 15	
Sex, male, *n* (%)	23	(77)	11	(73)	12	(80)
Age, years	59	(54–75)	57	(52–78)	62	(57–69)
SOFA score	10	(9–14)	13	(10–15)	10	(8–12)
Septic shock, *n* (%)	19	(63)	10	(67)	9	(60)
Time to PLR test from ICU admission, days	3	(3–13)	3	(2–13)	4	(3–13)
Hemoglobin, gr/dL	8.5	(8.0–10.5)	8.5	(8.0–11.0)	8.4	(7.7–9.4)
IVC diameter, mm	20	(17–23)	19	(17–23)	21	(16–23)
CVP, mmHg	12	(9–14)	11	(8–17)	13	(9–14)
VTI, cm	14.2	(13.0–17.4)	15.1	(13.5–19.5)	13.8	(12.8–17.4)
MAP, mmHg	77	(73–83)	76	(73–83)	77	(73–88)
Heart rate, beats/min	91	(85–99)	89	(83–106)	92	(85–99)
pH	7.35	(7.30–7.42)	7.35	(7.30–7.42)	7.35	(7.30–7.46)
PaCO_2_, mmHg	40	(37–43)	40	(36–43)	41	(37–44)
PcvCO_2_, mmHg	46	(42–50)	44	(41–50)	48	(43–51)
CaO_2_, mL/dL	11.	1(10.5–13.5)	11.1	(10.6–14.6)	11.1	(10.1–12.4)
CvO_2_, mL/dL	8.6	(7.8–11.4)	9.3	(7.8–11.7)	8.5	(7.8–9.5)
Lactate, mmol/L	1.9	(1.0–3.1)	1.9	(1.0–3.9)	1.5	(0.9–3.1)
Noradrenaline dose, µg/kg/min	0.19	(0.03–0.37)	0.22	(0.17–0.41)	0.04	(0.01–0.3)
PaO_2_, mmHg	118	(99–137)	112	(91–140)	122	(106–137)
PcvO_2_, mmHg	45	(41–50)	45	(38–51)	43	(41–49)
SaO_2_, %	98	(97–98)	98	(96–98)	98	(97–98)
VTI change, %	10	(7–18)	7	(7–8)	18	(13–25)
C(a-cvO_2_), mL/dL	2.7	(1.7–3.3)	2.5	(1.7–3.3)	2.8	(1.7–3.7)
P(cv-a)CO_2_/C(a-cv)O_2_, mmHg · dL/mLO_2_	2.4	(2.0–2.9)	2.2	(1.2–2.8)	2.5	(2.1–3.3)
P(cv-a)CO_2_, mmHg	6.4	(5.1–7.4)	6.4	(4.7–7.3)	6.6	(5.7–7.8)
ΔP(cv-aCO_2_)/C(a-cv)O_2_, mmHg · dL/mLO_2_	0.22	(−0.39–0.17)	0.10	(−0.15–0.57)	−0.38	(−0.97–−0.34) ^#^
ΔP(cv-a)CO_2_, mmHg	−0.3	(−1.6–0.7)	0.6	(−0.1–1.1)	−1.3	(−2.0–−0.6) ^#^
Respiratory rate, breaths/min	24	(22–26)	24	(22–26)	24	(22–26)
V_T_, ml	470	(430–500)	470	(397–535)	460	(430–500)
Minute ventilation, L/min	10.7	(9.6–12.2)	12.0	(9.0–13.3)	10.6	(9.8–11.0)
PEEP, cm H_2_O	10	(8–13)	10	(8–14)	10	(8–12)
PaO_2_/FiO_2_, mmHg	264	(189–305)	230	(178–286)	290	(212–340)
Driving pressure, cm H_2_O	10	(9–12)	9	(8–11)	11	(9–13)

Data are expressed as median (interquartile range), unless otherwise defined. ^#^ Significant differences between preload responders and preload non-responders (*p* < 0.001). All other variables were non-significant. * patients with an increase in VTI of ≥10% during the PLR maneuver. Abbreviations: PLR, passive leg raising; MAP, mean arterial pressure; SOFA, sequential organ failure assessment; IVC, inferior vena cava; CVP, central venous pressure; VTI, velocity-time integral; V_T_, tidal volume; PEEP, positive end-expiratory pressure; P(cv-a)CO_2_, central venous minus arterial carbon dioxide partial pressures; C(a-cv)O_2_, difference in arterial-to-central venous oxygen contents; Δ, difference between PLR and pre-PLR maneuver.

**Table 2:** Spearman correlation coefficient matrix for CO_2_-derived variables by ΔVTI and IVC, CVP, Lactate, SOFA MAP, and HR.

	ΔVTI	ΔP(cv-a)CO_2_	ΔP(cv-aCO_2_) /C(a-cv)O_2_	IVC	CVP	Lactate	SOFA	MAP	HR
ΔVTI		−0.601 **	−0.584 **	0.113	0.361	−0.149	−0.248	0.132	−0.160
ΔP(cv-a)CO_2_	−0.601 **		0.857 **	−0.291	−0.182	0.027	0.430	0.290	−0.111
ΔP(cv-aCO_2_)/C(a-cv)O_2_	−0.584 **	0.857 **		−0.418 *	−0.234	−0.089	0.223	0.282	−0.170
IVC	0.113	−0.291	−0.418 *		0.461 *	0.478 *	0.091	−0.214	0.262
CVP	0.361	−0.182	−0.234	0.461 *		0.494 **	0.473 *	0.043	0.239
Lactate	−0.149	0.027	−0.089	0.478 *	0.494 **		0.632 **	0.012	0.573 **
SOFA	−0.248	0.430	0.223	0.091	0.473 *	0.632 **		0.040	0.275
MAP	0.132	0.290	0.282	−0.214	0.043	0.012	0.040		−0.003
HR	−0.160	−0.111	−0.170	0.262	0.239	0.573 **	0.275	−0.003	

* *p* < 0.01, ** *p* < 0.001. Abbreviations: VTI, velocity-time integral; P(cv-a)CO_2_, central venous minus arterial carbon dioxide partial pressures; C(a-cv)O_2_, difference in arterial-to-central venous oxygen contents; MAP, mean arterial pressure; SOFA, sequential organ failure assessment; IVC, inferior vena cava; CVP, central venous pressure; HR, heart rate; Δ, difference between PLR and pre-PLR maneuver.

**Table 3:** Hemodynamic and CO_2_-derived markers dynamics across the PLR test.

Variable	Pre-PLR	PLR	Post-PLR
VTI	14.2 (13.0–17.4)	17.0 (15.5–19.1) *	14.8 (12.5–18.0)
P(cv-a)CO_2_	6.45 (5.1–7.4)	5.80 (4.6–7.1)	5.7 (4.3–7.4)
P(cv-a)CO_2_/C(a-cv)O_2_	2.4 (2.0–2.9)	2.1 (1.9–2.1)	2.2 (1.8–3.0)
MAP	77 (73–83)	84 (75–90) *	77 (70–86)
Heart rate	91 (85–99)	90 (82–98)	89 (82–100)

* *p* < 0.001 for: PLR vs. pre-PLR and PLR vs. post-PLR test. All data are presented as median (IQR). The overall trend over time was estimated with Friedman test, while the between-groups comparisons were performed by Mann–Whitney test. Abbreviations: PLR, passive leg raising; VTI, velocity-time integral; P(cv-a)CO_2,_ central venous minus arterial carbon dioxide partial pressures; C(a-cv)O_2_, difference in arterial-to-central venous oxygen contents; MAP, mean arterial pressure.

**Table 4:** Multivariable Firth’s penalized logistic regression analyses with bootstrap confidence intervals.

Variable	OR	95% CI	*p* Value	95% Bootstrap CI
*Model 1*				
CVP	1.01	0.86–1.21	0.93	0.66–1.31
Lactate	0.95	0.36–1.18	0.66	0.27–2.36
ΔP(cv-a)CO_2_	0.48	0.20–0.89	0.016	0.00–0.85
Sensitivity analysis 1: Model 1 plus noradrenaline dose				
ΔP(cv-a)CO_2_	0.50	0.21–0.92	0.023	0.00–0.97
Sensitivity analysis 2: Model 1 plus shock category (septic vs. non-septic)				
ΔP(cv-a)CO_2_	0.49	0.20–0.90	0.018	0.00–0.87

Odds ratios and *p*-values are derived from Firth penalized logistic regression. Bootstrap confidence intervals were estimated using 1000 nonparametric resamples and are presented as a sensitivity analysis. Differences between confidence interval estimates reflect the limited sample size. Abbreviations: Δ, difference between PLR and pre-PLR test; P(cv-a)CO_2,_ central venous minus arterial carbon dioxide partial pressures; CVP, central venous pressure.

**Table 5:** Performance characteristics of the CO_2_-derived indices.

	Threshold	Sensitivity	Specificity	PPV	NPV	PLR	NLR
ΔP(cv-a)CO_2_	−0.25	0.93 (0.68–0.99)	0.93 (0.68–0.99)	0.93 (0.68–0.99)	0.93 (0.68–0.99)	14 (2.10–44.71)	0.07 (0.02–0.48)
ΔP(cv-a)CO_2_/ C(a-cv)O_2_	−0.003	0.78 (0.49–0.95)	0.93 (0.68–0.99)	0.91 (0.61–0.99)	0.82 (0.56–0.96)	11.78 (1.74–38.48)	0.23 (0.10–0.65)

95% confidence intervals are within parentheses. Abbreviations: Δ, difference between PLR and pre-PLR; P(cv-a)CO_2_, central venous minus arterial carbon dioxide partial pressure; C(a-cv)O_2_, arterial minus central venous oxygen content; PPV, positive predictive value; NPV, negative predictive value; PLR, positive likelihood ratio; NLR, negative likelihood ratio.

## Data Availability

The data used or analyzed in the present study are available from the corresponding author on reasonable request.

## Supplementary Materials

The following supporting information can be downloaded at: https://www.mdpi.com/article/10.3390/jcm15041551/s1, Figure S1: Bland-Altman plot for velocity-time integral measurements (VTI); Figure S2: Calibration plot of the primary Firth logistic regression model.
