# Supplementary material for: Changes in CO2-Derived Variables, Induced by Passive Leg Raising Test, Detect Preload Responsiveness in Mechanically Ventilated Patients: A Pilot Study
Source: J Clin Med. 2026 Feb 15;15(4):1551. doi: 10.3390/jcm15041551 (PMC12941753; doi:10.3390/jcm15041551)
Supplement: Supplementary file 1 [file jcm-15-01551-s001.zip › jcm-4095654-supplementary.pdf]

SUPPLEMENT 1

Changes in CO_2-Derived Variables, Induced by Passive Leg Raising Test, Predict Preload Responsiveness in Mechanically Ventilated Patients: a pilot study

Angeliki Baladima, Stelios Kokkoris, Dimitrios Tzalas, Constantina Kolonia, Theodora Ntaidou, Theodoros Pittaras, Athanasios Trikas, Ioannis Vasileiadis and Christina Routsi

METHODS

Characterization of the cohort in terms of ARDS severity and dead-space ventilation

ARDS severity was categorized according to PaO_2/FiO_2 ratio: Severe (<100): 7%, moderate (101-200): 20%, mild (201-300): 44% of patients. Increased dead space ventilation was defined as a ventilatory ratio (VR) value > 2, and was found in 25% of patients.

VR was calculated as: *VR= (VE measured X $PaCO_2$ measured) / (VE predicted X 37.5)*, where "VE measured" is the measured minute ventilation (mL/min), "$PaCO_2$ measured" is the measured arterial pressure of carbon dioxide (mmHg), "VE predicted" is the predicted minute ventilation calculated as predicted body weight X 100 (mL/min). The predicted body weight of male patients was calculated as equal to 50 + 0.91(centimeters of height – 152.4); that of female patients was calculated as equal to 45.5 + 0.91(centimeters of height – 152.4) (1). "$PaCO_2$ ideal" is the expected $PaCO_2$ in normal lungs if ventilated with the predicted VE. $PaCO_2$ ideal is set as 37.5 mmHg for all patients. VR is a unitless ratio, and a value approximating 1 would represent normally ventilated lungs.

1. *The Acute Respiratory Distress Syndrome Network. Brower RG, Matthay MA, Morris A, Schoenfeld D, Thompson BT, Wheeler A. Ventilation with lower tidal volumes as compared with traditional tidal volumes for acute lung injury and the acute respiratory distress syndrome. N Engl J Med. 2000;342:1301–08. https://doi.org/10.1056/NEJM200005043421801*

Figure S1. Bland-Altman plot for velocity-time integral measurements (VTI).

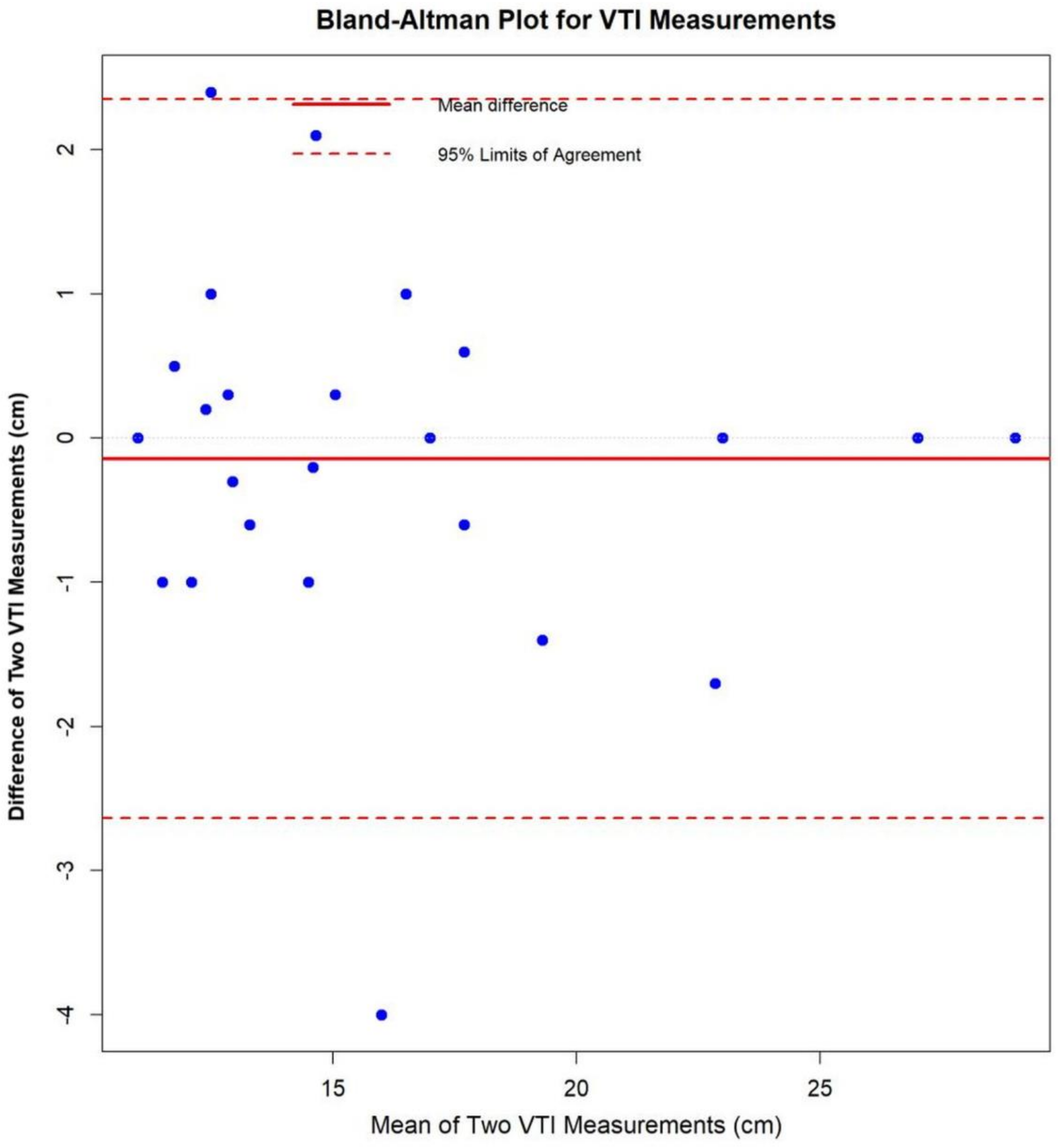

Figure S2. Calibration plot of the primary Firth logistic regression model. The plot shows predicted probabilities versus observed outcomes for the primary penalized logistic regression model. The dashed diagonal line represents perfect calibration. The blue curve depicts a Loess-smoothed calibration estimate, and black points represent individual patients. The model demonstrates reasonable calibration at low predicted probabilities, with underestimation of risk at intermediate probabilities and increased uncertainty at the extremes, consistent with the small sample size.

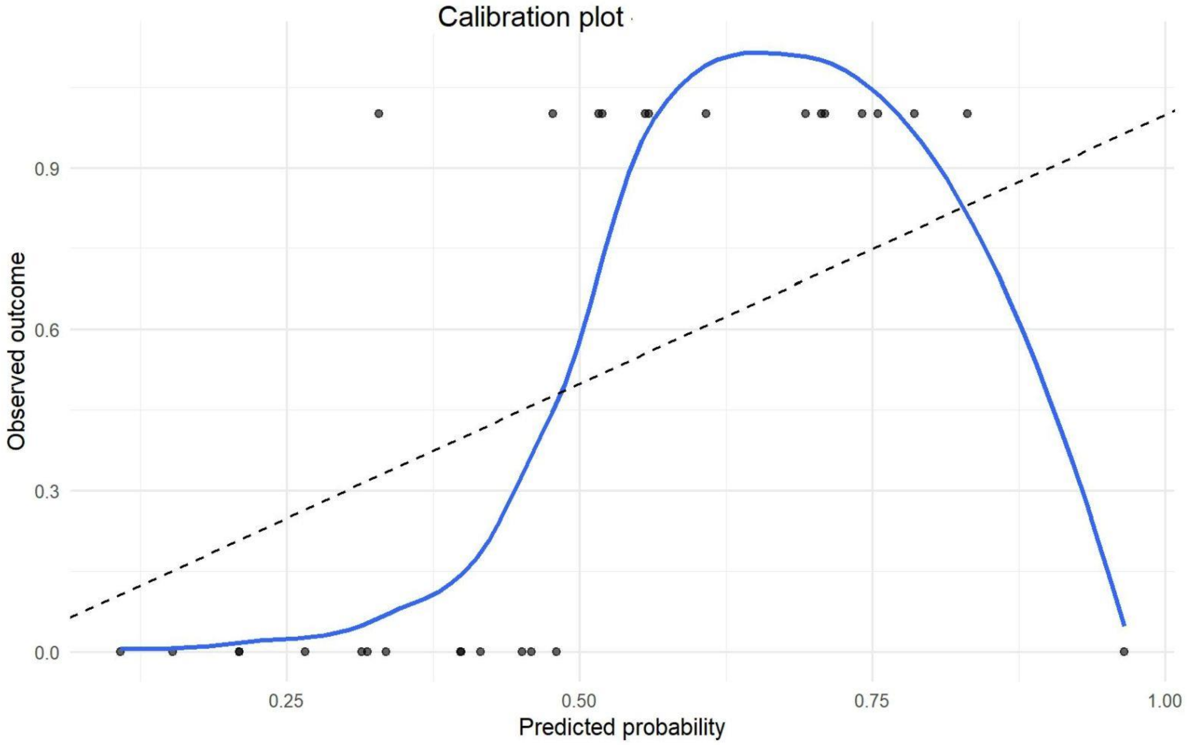

SUPPLEMENT 2

Changes in CO_2-Derived Variables, Induced by Passive Leg Raising Test, Detect Preload Responsiveness in Mechanically Ventilated Patients: a pilot study

Angeliki Baladima, Stelios Kokkoris, Dimitrios Tzalas, Constantina Kolonia, Theodora Ntaidou, Theodoros Pittaras, Athanasios Trikas, Ioannis Vasileiadis and Christina Routsi

Calculation example for the arterial oxygen content (CaO_2), central venous oxygen content ($CcvO_2$) and $P_{(cv-a)}CO_2$ / $C(a-cv)O_2$ ratio

1. **CaO_2** can be calculated by the formula: CaO_2 = (SaO_2 x Hb x1.34) + (0.0031x PaO_2)
This formula calculates the ml of O_2 per dL of blood

Example for a healthy person: SaO_2=100%, Hb=15g/dL, 1.34 is a constant in mlO_2/g Hb, PaO_2=100 mmHg, 0.0031 is a constant in ml/mmHg/dL

Therefore:

CaO_2 = (1.00 x 15g/dL x 1.34 mlO_2/g) + (0.0031 ml/mmHg/dL x 100 mmHg)

=20.1 mlO_2/dL +0.3 mlO_2/dL = 20.4 mlO_2/dL of blood or simply **20.4ml/dL**

2. **$CcvO_2$** can be calculated by the formula: $CcvO_2$ = ($ScvO_2$ x Hb x 1.34) + (0.0031x $PcvO_2$)

Example for a healthy person: $ScvO_2$=75%, Hb=15g/dL, 1.34 is a constant in mlO_2/g Hb, $PcvO_2$=40 mmHg, 0.0031 is a constant in ml/mmHg/dL

Therefore:

$CcvO_2$ = (0.75 x 15g/dL x 1.34 mlO_2/g) + (0.0031ml/mmHg/dL x 40 mmHg)

=15.08 mlO_2/dL + 0.12 mlO_2/dL = 15.2 mlO_2/dL of blood or simply **15.2 ml/dL**

3. Subsequently, the $C(a-cv)O_2$ can be calculated as CaO_2 – $CcvO_2$ in ml/dL

4. Finally, the central venous minus arterial carbon dioxide partial pressures ($P_{(}cv\text{-}a_{)}CO_2$) can be calculated in mmHg and therefore, the $P_{(}cv\text{-}a_{)}CO_2$ / $C(a\text{-}cv)O_2$ ratio can be calculated in mmHg · dL/ml

References

1. Mekontso-Dessap A, Castelain V, Anguel N, Bahloul M, Schauvliege F, Richard C, et al. Combination of venoarterial PCO2 difference with arteriovenous O2 content difference to detect anaerobic metabolism in patients. Intensive Care Med. 2002;28:272–7.

2. Mesquida J, Saludes P, Gruartmoner G, et al. Central venous-to-arterial carbon dioxide difference combined with arterial-to-venous oxygen content difference is associated with lactate evolution in the hemodynamic resuscitation process in early septic shock. Crit. Care 2015; 19:126
